# The Impact of Hyperkalemia on Mortality and Healthcare Resource Utilization Among Patients With Chronic Kidney Disease: A Matched Cohort Study in China

**DOI:** 10.3389/fpubh.2022.855395

**Published:** 2022-03-24

**Authors:** Jiahui Zhang, Xiaoning He, Jing Wu

**Affiliations:** ^1^School of Pharmaceutical Science and Technology, Tianjin University, Tianjin, China; ^2^Center for Social Science Survey and Data, Tianjin University, Tianjin, China

**Keywords:** chronic kidney disease, hyperkalemia, mortality, healthcare resource utilization, costs

## Abstract

**Objectives:**

This study aimed to estimate the impact of hyperkalemia on 1-year survival and all-cause healthcare resource utilization among patients with chronic kidney disease in China.

**Methods:**

Adult new-onset chronic kidney disease patients were identified between 2012 and 2016, among which the hyperkalemia and non-hyperkalemia cohorts were further selected and matched. Survival and all-cause healthcare resource utilization during a 12 month period were compared using Kaplan-Meier curves with log-rank test, Cox proportional hazard model, and Kaplan-Meier sample average method.

**Results:**

Among 1,003 pairs of patients (mean age 67.2 ± 14.3 years), the 1-year all-cause mortality was 5.39-times higher in the hyperkalemia cohort than the non-hyperkalemia cohort (hazard ratio = 5.39, *P* < 0.001). The mean costs and number of healthcare services among the hyperkalemia patients were significantly higher (*P* < 0.05) in each follow-up month. An annual increase cost of ¥38,479 was observed in the hyperkalemia cohort, largely due to the sharp increase in inpatient costs during the first month after hyperkalemia events (¥22,204 vs. 1,032, *P* < 0.001). Medication costs were the most important cost component for both cohorts [¥26,786 (45%) vs. ¥12,378(60%)]. However, hyperkalemia patients spent more on non-medication treatment [¥13,410(23%) vs. ¥2,335(11%)] including nursing, monitoring, etc. Hyperkalemia patients had more annual inpatient admissions (1.9 vs. 0.7) and length of stays (28.6 vs. 8.7), while the number of outpatient visits (36.8 vs. 36.4) were similar.

**Conclusions:**

In Chinese chronic kidney disease patients, hyperkalemia is associated with substantially increased clinical and economic burdens that are driven by the short period following the hyperkalemia events, which strengthens calls for sufficient supervision and management of serum potassium.

## Introduction

Hyperkalemia (HK), defined as serum potassium (K^+^) >5.0 mmol/L, is an electrolyte abnormality that disrupts the cellular membrane potential and transmission of action potentials (1). Chronic kidney disease (CKD) is the most important condition that predisposes hyperkalemia, where the decreased glomerular filtration rate (GFR) and the presence of tubulointerstitial dysfunction diminishes the potassium metabolism (2). Other comorbidities related to the impaired circulation and metabolism could also increase the risk of HK, including heart failure (HF), diabetes mellitus (DM), and hypertension. Additionally, for patients with cardiorenal diseases, the essential use of renin–angiotensin–aldosterone system inhibitors (RAASi) may present a challenge in the management of K^+^ due to their mechanisms that suppress the critical medium of potassium excretion (3–7). Acute abnormal elevation of K^+^ levels may directly lead to muscle cramps, dyspnea, cardiac arrhythmias, and even sudden death (8–10). Meanwhile, the threat of HK also comes from the treatment requiring down-titration or even discontinuation of RAASi, which sacrifices the long-term cardiorenal benefits and has proven to be one of the major barriers to achieving optimal control of disease progression (5, 11).

For patients with CKD, given the high exposure risk and dilemma of management strategies, the exacerbated clinical and economic burdens related to HK were commonly reported in different disease conditions and populations including the United States (12, 13), England (14), Korea (15), Japan (16, 17) and Denmark (18), etc., while it has not been described in China. According to published studies, the estimated hazard ratios of increased mortality among patients with HK largely varies from 1.1 (12) to 17.7 (16), and the HK-associated annual cost increase also differed from $12,530 to 33,696 ($2,808 monthly), compared to patients without HK (16–18). The observable diversity of existing results indicates the necessity to investigate the short and long-term effects of HK across various regions due to the difference in the prevalence of risk factors, available treatment strategies, and overall healthcare level.

Additionally, in previous studies that examined the cost and healthcare resource utilization (HRU), patients who could not be followed up throughout the study period were usually excluded to capture the annual differences, while patients under the acute fatal threaten of HK may have been overlooked, and the effect of HK on the population would be inadequate. Alternatively, the comparison of costs would be biased by the different length of follow-up period between patients with and without HK because of the influence of HK. The survival-adjusted estimator, which weights the mean cost by the survival probability of each time interval, may be beneficial under this situation (19), but has been scarcely explored in this topic before.

This study aimed to address this evidence gap by estimating real-world associations of hyperkalemia and disease burdens in a large cohort of Chinese patients with new-onset CKD. Specifically, the study addressed two objectives: (1) to investigate the associations between hyperkalemia and all-cause mortality; and (2) to assess the influence of HK on economic burdens by considering survival conditions, including all-cause healthcare resource utilization (HRU) and direct medical costs.

## Methods

### Data Source

The data for this retrospective cohort study were collected from the Urban Employee Basic Medical Insurance (UEBMI) claims of Tianjin. The UEBMI is the basic medical insurance plan for employees (including retirees) in both public and private enterprises and covers 50.8% of registered Tianjin residents. The UEBMI database provides longitudinal views of patient-level eligibility, demographics, prescription claims, and inpatient and outpatient claims (diagnosis, date of visit, information of hospitals, etc.) with related cost information (type of service, amount, price, data of consumption, etc.). Information about death is recorded separately and could be linked by the unique identity codes. Data in UEBMI from 2011 to 2017 were available at the time of this study and a random sample of all enrollees and related records were extracted for this retrospective observational analysis. Ethical approval of this study was obtained from the Ethics Committee, School of Pharmaceutical Science and Technology, Tianjin University.

### Study Population

The index period from January 1, 2012, to December 31, 2016, was defined in this study, with data being required from at least a 1-year time window to obtain the covariates and assess the outcomes, respectively. Adult patients (age ≥ 18 years) who were newly-diagnosed with CKD between the index period were first identified based on having ≥1 inpatient or outpatient visit, along with the diagnosis of CKD (ICD-10 codes E1x.2[x = 0-4], I12, I13, N03, N04, N05, N18, N19, Z49, Z99.2, supplemented by Chinese free texts). Furthermore, patients who experienced ≥1 inpatient or outpatient visit with a diagnosis of HK (ICD-10 code E87.5, supplemented by Chinese free texts) after being diagnosed with CKD between January 1, 2012, and December 31, 2016, were included in the HK cohort. The non-HK cohort was formed by patients who were free of HK diagnosis during the study period.

Patients in the non-HK cohort were matched with patients in the HK cohort through a 1:30 propensity score matching, based on sex and the year and age of being diagnosed as CKD. The index dates were defined as dates of the earliest identified HK event for patients in the HK cohort and, then, assigned to their counterparts in the non-HK cohort. The included patients were required to be continuously enrolled during the 12 months prior to (baseline period) and 12 months following the index date. Discontinued enrollment due to death was allowed. The follow-up period was accumulated from the index date to the discontinuation of enrollment, death, or December 31, 2017, whichever came first.

The propensity score for each patient was then derived using a logistic regression model based on the following baseline demographic and clinical characteristics: age, sex, duration of CKD, Charlson Comorbidity Index (CCI), presence of comorbidities, medication use, all-cause medical resource utilization, and direct medical cost. The balanced HK and non-HK cohorts were further selected by a 1:1 matching with the propensity score. Absolute standardized differences for matching covariates were examined to assess the pre- and post-match bias, with values under 10% considered to be inconsequential (20).

### Outcome Measures

Incidence of all-cause mortality was determined during the 12-month follow-up period and the period from index date to the end of the study. The all-cause HRU per patient was assessed by the percentages of patients who utilized inpatient and outpatient services, the numbers of hospitalizations and outpatient visits, and annualized length of stay (LOS). The LOS per admission was further calculated among the matched inpatients. All-cause medical costs were estimated during the 12-month follow-up period from the perspective of health care system—the co-payments by the insurers and patients. The total costs per patient were composed of costs of inpatient and outpatient services through relative claims, which were further attributed to costs of medications, examinations, non-medication treatments (e.g., nursing and monitoring, etc.), surgery, medical consumables, and other utilized medical services (e.g., blood transfusion, etc.). The costs per admission or per visit were also calculated among matched inpatients and outpatients, respectively.

### Covariates

Baseline covariates of interest were those that may influence the circulatory systems and metabolic actions. Baseline comorbidities included in this study were heart failure, diabetes mellitus, hypertension, dyslipidemia, cardiovascular disease (except for heart failure), peripheral vascular disease, and cerebrovascular disease. History of medication was reflected as prescriptions of RAASi, antidiabetic drugs, anti-dyslipidemia drugs, non-steroidal anti-inflammatory drugs, and other anti-hypertension drugs, such as β-blockers, calcium channel blockers, etc. Baseline HRU was presented by the number of outpatient and inpatient visits and length of stay.

### Data Analysis

All baseline characteristics and outcome measures were evaluated descriptively for each cohort. Continuous variables were summarized as means and standard deviations, while categorical variables were reported as frequencies and percentages. The comparisons between the HK and non-HK cohort were performed by Student's *t*-test or χ^2^-test, where appropriate. No further adjustment was presented in the analysis of mortality, HRU, and costs, as the patient characteristics of the HK and non-HK cohorts were balanced through matching. The Kaplan-Meier method and log-rank test were used to describe and compare the overall survival for the HK and non-HK cohort, respectively, during the entire 12-month follow-up period. Hazards ratios (HRs) were calculated by Cox proportional hazard models to ascertain the association between HK and all-cause mortality.

Kaplan-Meier sample average (KMSA) method was used to evaluate the differences in the HRU and costs between the HK and non-HK cohorts accounting for the discrepancy of 12-month survival related with HK. Specifically, the probability of surviving to each time interval (i.e., post-index month) was initially extracted from the Kaplan-Meier survival curves. Then the probability of surviving to the start of each time interval was multiplied by the HRU and costs incurred during the corresponding time interval conditional on surviving to the start of that interval. Finally, the point estimate of annual all-cause HRU and costs was obtained by summarizing the average monthly cost for each cohort (12 months total), and a bootstrap approach was used to estimate the 95% confidence intervals (CIs), where 1,000 iterations with 500 pairs of matched HK patients and controls were sampled.

To avoid the possible bias from extreme health status, sensitivity analysis was conducted among matched patients without any diagnosis of malignant disease during the study period and patients who survived the 12-month follow-up period, respectively. Two-sided *P*-values of < 0.05 were considered statistically significant. All analyses were performed using STATA 13.1 (StataCorp, Texas, U.S).

## Results

### Baseline Characteristics

A total of 207,232 patients with new-onset CKD were identified between 2012 and 2016, among which 1,321 patients experienced at least one episode of HK after diagnosis of CKD, and 1,003 pairs of HK patients and matched controls were finally included. Table 1 shows that patients in both cohorts had a mean age of 67 years and mean CKD duration of 13 months. The mean CCI score was estimated as 3.9, with hypertension (79.4% in HK cohort vs. 81.9% in non-HK cohort) being the most common comorbidity. Concomitant medications were correspondingly frequent in both cohorts, with half of the patients having prescriptions of angiotensin receptor blockers (ARB), which is one of the most critical risk factors of HK. Compared to the patients in the non-HK cohort, the patients in the HK cohort were in a worse health status (Supplementary Table 1), but the demographic and clinical characteristics were not significantly different between the cohorts because of matching (Table 1).

**Table 1:** Baseline characteristics of the HK and non-HK cohorts after propensity score matching.

**Variables**	**HK cohort** ***N*** **= 1,003**		**Non-HK cohort** ***N*** **= 1,003**		***p*** **-value**	**Standardized difference**
	**Mean/n**	**SD/%**	**Mean**	**SD/%**		
**Demographic characteristics**						
Age at index date [Mean (SD)]	67.4	14.4	66.9	14.2	0.409	0.04
Female [*n* (%)]	407	40.6%	404	40.3%	0.891	0.01
**CCI**	3.9	2.6	3.9	2.3	0.943	0.00
CKD duration [month, mean(SD)]	12.7	15.3	12.8	13.5	0.767	−0.01
**Baseline comorbidity [*****n*** **(%)]**						
Hypertension	796	79.4%	821	81.9%	0.158	−0.06
Dyslipidemia	465	46.4%	499	49.8%	0.129	−0.07
T2DM	440	43.9%	428	42.7%	0.589	0.02
Heart failure	204	20.3%	188	18.7%	0.368	0.04
CVD						
Arrhythmias	280	27.9%	285	28.4%	0.804	−0.01
Angina	208	20.7%	193	19.2%	0.402	0.04
Myocardial Infarction	98	9.8%	105	10.5%	0.604	−0.02
Other CVD	389	38.8%	411	41.0%	0.316	−0.04
CBD						
Stroke	309	30.8%	331	33.0%	0.292	−0.05
Transient Ischemic Attack	35	3.5%	39	3.9%	0.636	−0.02
Other CBD	121	12.1%	125	12.5%	0.785	−0.01
PVD	155	15.5%	168	16.7%	0.430	−0.04
Peptic ulcer	102	10.2%	93	9.3%	0.498	0.03
COPD	60	6.0%	68	6.8%	0.465	−0.03
Rheumatic disease	47	4.7%	52	5.2%	0.606	−0.02
**Baseline treatment [*****n*** **(%)]**						
Dialysis	21	2.1%	13	1.3%	0.008	0.06
**RAASi prescription**						
ARB	482	48.1%	494	49.3%	0.592	−0.02
MRA	309	30.8%	324	32.3%	0.471	−0.03
ACEi	232	23.1%	235	23.4%	0.874	−0.01
**Non-RAASi prescription**						
Calcium channel blockers	639	63.7%	664	66.2%	0.242	−0.05
Loop Diuretics	540	53.8%	562	56.0%	0.324	−0.04
NSAID	537	53.5%	539	53.7%	0.929	0.00
Anti-platelet	475	47.4%	493	49.2%	0.421	−0.04
β-blocker	425	42.4%	450	44.9%	0.260	−0.05
Anti-dyslipidemia	392	39.1%	407	40.6%	0.494	−0.03
Insulin	374	37.3%	382	38.1%	0.712	−0.02
OAD	288	28.7%	279	27.8%	0.655	0.02
Bronchodilators	274	27.3%	276	27.5%	0.920	0.00
Anti-hypertension	231	23.0%	227	22.6%	0.832	0.01
**All-cause healthcare resource utilization and medical costs per patient**						
Number of hospitalizations	1.3	1.7	1.3	2.2	0.865	−0.01
Length of stay	18.1	30.7	16.8	30.7	0.355	0.04
Number of outpatient visits	35.9	35.3	35.8	32.2	0.939	0.00
All-cause direct medical cost (¥)	33,015	43,676	31,353	45,046	0.402	0.04

Standard difference was used to compare balance in measured variables between HK and non-HK subjects in the matched sample with that in the unmatched sample. It was defined as d = (X_HK_-X_non−HK_)/(($S_{HK}^{2}$-$S_{non-HK}^{2}$)/2)^∧^0.5) for continuous variables (where X denote the sample mean of the covariate in HK and non-HK subjects, and S^2^ denote the sample variance, respectively) and d = (P_HK_-P_nonHK_)/((( P_HK_^*^(1- P_HK_)+ P_non−HK_^*^(1- P_non−HK_))/2)^∧^0.5) for dichotomous variables (where p denote the proportion of the covariate in HK and non-HK subjects). Imbalance defined as absolute value >0.1. SD, Standard Deviation; CCI, Charlson Comorbidity Index; CKD, Chronic Kidney Disease; HF, Heart Failure; T2DM, Type 2 Diabetes Mellitus; CVD, Cardiovascular Disease; CBD, Cerebrovascular Disease; PVD, Peripheral Vascular Disease; COPD, Chronic Obstructive Pulmonary Disease; ACEi, Angiotensin-Converting Enzyme Inhibitors; ARB, Angiotensin Receptor Blockers; MRA, Mineralocorticoid Receptor Antagonists; OAD, Oral Antidiabetic Drugs; NSAID, Non-Steroidal Anti-Inflammatory Drugs.

### Mortality of HK and Non-HK Cohorts

**Figure 1:**
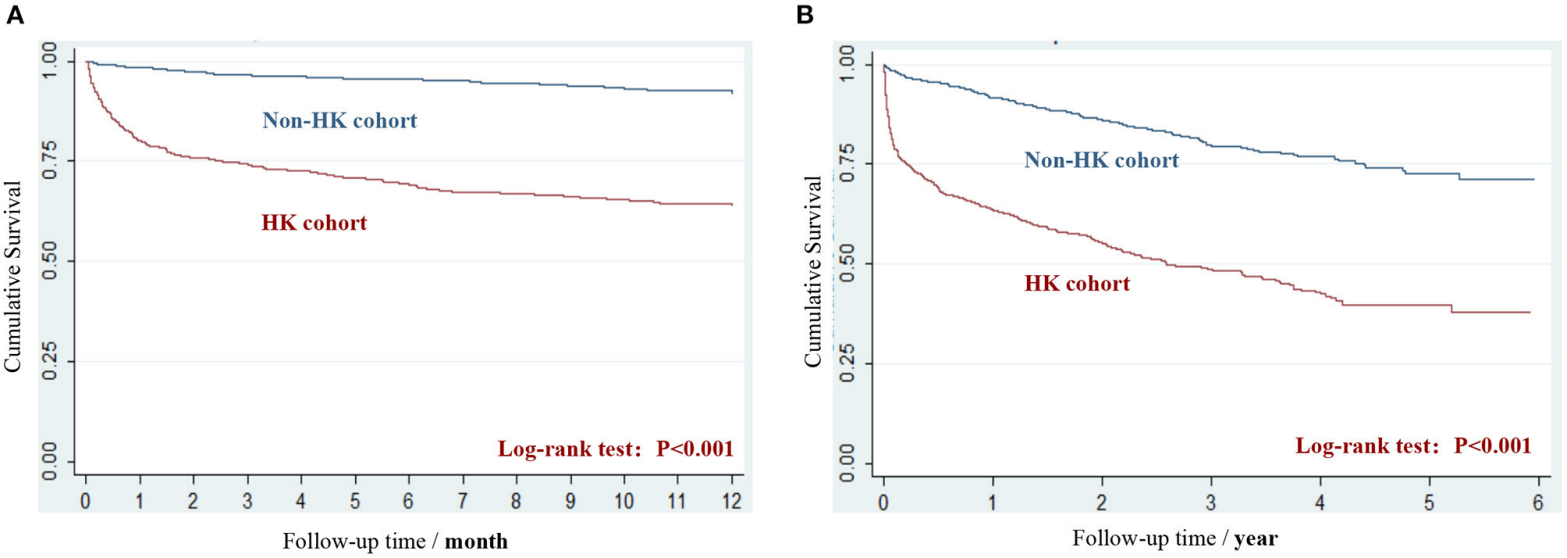
The Kaplan-Meier survival curves of HK and non-HK cohort. **(A)** Survival during the 12-month follow-up period. **(B)** Survival during the period from index date to the end of study.

A significantly negative impact of HK on cumulative survival time (20.4 ± 18.0 vs. 31.1 ± 16.8 months, *P* < 0.001) during the 25.7 ± 18.2 months of the follow-up period was observed, and the difference (log-rank test, *P* < 0.001) of overall survival status during the 12-month follow-up period and the total follow-up period is presented in Figures 1A,B, respectively. Within the 12 months after the index date, 36.4% of patients in HK and 8.3% of patients in non-HK cohorts died, and a pronounced increased risk was observed with a HR of 5.4 (95% CI 4.2–6.8; Table 2). The difference of mortality between the two cohorts was narrowed, but was still significant during the ongoing follow-up period, with an overall 3.7 times (49.5 vs. 18.1%, 95% CI: 3.2–4.4; Table 2) higher risk exhibited in the HK cohort.

**Table 2:** Mortality of the HK and non-HK cohorts during the follow-up period.

	**HK cohort**	**Non-HK cohort**	***p*** **-value**	**HR**	**95% CI**
Mortality in the 12-month follow-up period	36.4%	8.3%	<0.001	5.4	[4.2, 6.8]
Mortality in the total follow-up period	49.5%	18.1%	<0.001	3.7	[3.2, 4.4]

*HR, hazard ratio; CI, confidence interval*.

### Healthcare Resource Utilization of HK and Non-HK Cohorts

Compared to the non-HK cohort, more patients in the HK cohort (86.5 vs. 32.1%, *P* < 0.001) experienced at least one hospitalization during the 12 months of follow-up period (including the index events). With the KMSA estimator applied, the patients in the HK cohort had one more hospitalization (1.9 vs. 0.7, *P* < 0.001) on an average and stayed 20 more days at the hospital (28.6 vs. 8.7, *P* < 0.001). Among the 288 pairs of matched patients with at least one hospitalization, the higher length of stay (15.8 vs. 12.7, *P* < 0.001) per admission was observed in the HK cohort. For the outpatient services of total cohorts, smaller number of patients (88.0 vs. 96.2%, *P* < 0.001) in the HK cohort experienced at least one outpatient visit, and there was almost no difference in number of outpatient visits per patient (36.8 vs. 36.4) in the HK and non-HK cohort (Table 3).

**Table 3:** The all-cause healthcare resource utilization and associated costs during the 12-month follow-up period.

	**HK cohort**	**Non-HK cohort**	**Difference**	***p*** **-value**
**Inpatient services of total patients (*****N*** **= 1,003 for both cohorts)**^***a***^				
Patients with at least 1 hospitalization	86.50%	32.10%	54.4%	<0.001
Number of admissions per patient [mean (95%CI)]^b^	1.9 (1.8–2.1)	0.7 (0.5–0.9)	1.2	<0.001
Total length of stay per patient [day, mean (95%CI)]	28.6 (25.0–31.7)	8.7 (6.5–10.5)	19.9%	<0.001
**Inpatient services of matched patients with hospitalizations (*****N*** **= 288 for both cohorts)**				
Number of admissions [mean ± SD (median)]	2.1 ± 1.5 (1.5)	2.1 ± 3.1 (1.0)	0	0.946
Length of stay per admission [day, mean ± SD (median)]	15.8 ± 13.5 (13.0)	12.7 ± 11.8 (11.0)	3.1	<0.001
Direct medical cost per admission [¥, mean ±SD (median)]	24,041 ± 28,975 (14,845)	16,637 ± 22,429 (11,205)	7,404	<0.001
**Outpatient services of total patients (*****N*** **= 1,003 for both cohorts)**				
Patient with at least 1 outpatient visit	88.00%	96.20%	−8.2%	<0.001
Number of visits per patient [mean (95%CI)]	36.8 (33.0–40.8)	36.4 (33.4–39.4)	0.4	<0.001
**Outpatient services of matched patients with outpatient visits (*****N*** **= 849 for both cohorts)**				
Number of visits per patient [mean ± SD (median)]	41.0 ± 48.2 (24)	37.7 ± 34.9 (28)	3.3	0.109
Direct medical cost per visit [¥, mean ±SD (median)]	753 ± 812 (479)	350 ± 566 (227)	403	<0.001
**All-cause direct medical costs (**¥, ***N*** **= 1,003 for both cohorts)**				
Total all-cause direct medical costs [mean (95%CI)]	59,120 (55,279–63,639)	20,732 (18,606–23,029)	38,479	<0.001
Medication [mean (%)]	26,696 (45%)	12,378 (60%)	14,318	<0.001
Examination [mean (%)]	8,955 (15%)	2,621 (13%)	6,334	<0.001
Treatment [mean (%)]	13,410 (23%)	2,335 (11%)	11,074	<0.001
Surgery [mean (%)]	592 (1%)	243 (1%)	348	<0.001
Medical Consumable [mean (%)]	5,357 (9%)	1,870 (9%)	3,487	<0.001
Other [mean (%)]	4,111 (7%)	1,284 (6%)	2,827	<0.001
Inpatient all-cause direct medical costs [mean (95%CI)]	41,855 (38,297–45,830)	10,980 (9,034–13,061)	30,875	<0.001
Medication [mean (%)]	17,179 (41%)	4,406 (40%)	12,773	<0.001
Examination [mean (%)]	7,757 (18%)	1,935 (18%)	5,822	<0.001
Treatment [mean (%)]	7,431 (18%)	1,551 (14%)	5,880	<0.001
Surgery [mean (%)]	552 (1%)	204 (2%)	348	<0.001
Medical consumable [mean (%)]	4,926 (12%)	1,660 (15%)	3,266	<0.001
Other [mean (%)]	4,010 (10%)	1,225 (11%)	2,786	<0.001
Outpatient all-cause direct medical costs [mean (95%CI)]	17,355 (15,755- 19,031)	9,752 (9,017- 10,498)	7,604	<0.001
Medication [mean (%)]	9,607 (55%)	7,972 (82%)	1,635	<0.001
Examination [mean (%)]	1,198 (7%)	686 (7%)	512	<0.001
Treatment [mean (%)]	5,979 (34%)	785 (8%)	5,194	<0.001
Surgery [mean (%)]	40 (0%)	40 (0%)	0	0.12
Medical Consumable [mean (%)]	430 (3%)	210 (2%)	220	<0.001
Other [mean (%)]	102 (1%)	59 (1%)	43	<0.001

a *The Index Hospitalizations Were Included*.

b *Mean (95% CI) for Variables Estimated by KMSA Method, Mean ± Standard Deviation (Median) for Other Variables*.

### Direct Medical Cost of HK and Non-HK Cohorts

The incremental total cost of ¥38,479 per patient during the 12-month follow-up period was observed in the HK cohort, which was primarily driven by higher inpatient costs (difference estimated as ¥30,875, *P* < 0.001). Outpatient costs (difference estimated as ¥7,604, *P* < 0.001) were also higher in the HK cohort than the non-HK cohort (Table 3). In addition, mean inpatient and outpatient costs per visit were around 1.4 and 2 times higher, respectively, in the HK cohort during the 12 months (inpatient, ¥24,041 ± 28,975 vs. 16,637 ± 22,429, *P* < 0.001; outpatient, 753 ± 812 vs. ¥350 ± 566, *P* < 0.001).

The increased annual total direct medical costs of patients in the HK cohort were mainly attributed to higher costs of medication (¥17,179 vs. 4,406, *P* < 0.001), non-medication treatments (¥7,431 vs. 1,551, *P* < 0.001), and examination (¥7,757 vs. 1,935, *P* < 0.001) incurred in hospitalization (Table 3). Costs of non-medication treatments (¥5,975 vs. 785, *P* < 0.001) during outpatient visits also contributed to the difference of amounts and structures of total costs (Table 3), which was possibly due to more visits in emergency department and higher hospital tiers. Consequently, the treatment costs accounted for a higher percentage among the patients in the HK cohort, while the patterns of inpatient costs were almost the same between the two cohorts (Table 3).

**Figure 2:**
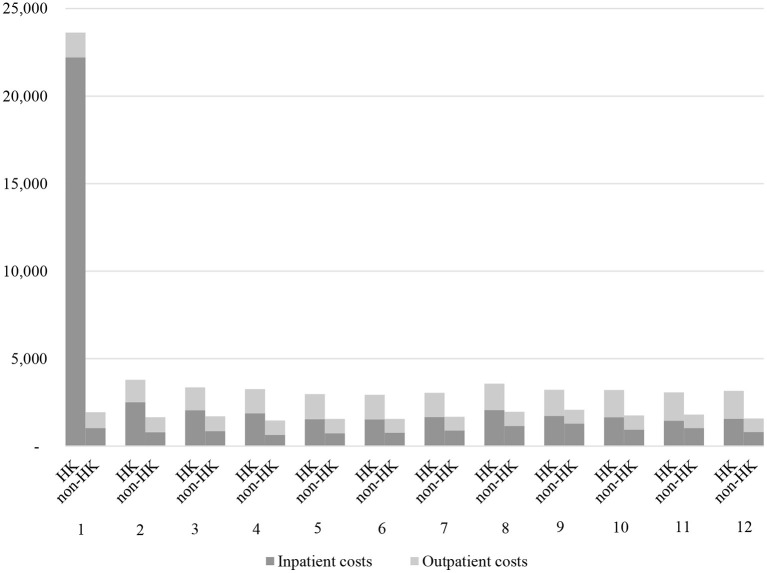
The monthly all-cause direct medical costs in the 12-month follow-up period (¥).

The difference of annual total cost per patient was dominated by the cost during the first month following the index date, which was further largely due to the sharp increase of inpatient costs after the HK events (¥22,204 vs. 1,032, *P* < 0.001, Figure 2; Supplementary Table 2). The outpatient costs for patients in both the cohorts were relatively stable, with only slight growth observed among patients in the HK cohort during each time intervals (Figure 2; Supplementary Table 2).

### Sensitivity Analysis

Sensitivity analysis showed that 633 pairs of patients remained in the study cohorts, when excluding patients with a diagnosis of malignant disease. The 3.7 and 5.1 times of risks of all-cause mortality in the HK cohort relative to the non-HK cohort during the whole and 12-month follow-up period were estimated, respectively, and the resulting difference in annual all-cause total costs was ¥40,856 (Table 4). In addition, there was an increase of ¥45,414 in annual all-cause total costs was observed among HK, based on 512 pairs of patients with ≥ 12 months of continuous enrollment after the index date (Table 4).

**Table 4:** The results of sensitivity analysis.

	**HK cohort**	**Non-HK cohort**	**Difference/HR**	***p*** **-value**
**Sensitivity analysis 1: Matched patients without any diagnosis of malignant disease**				
Mortality in the total follow-up period	44.00%	15.60%	3.73	<0.001
Mortality in the 12-month follow-up period	30.30%	6.80%	5.08	<0.001
Total all-cause direct medical costs (¥)	59,587	18,731	40,856	<0.001
Inpatient all-cause direct medical costs	41,048	9,461	31,587	<0.001
Outpatient all-cause direct medical costs	18,530	9,271	9,259	<0.001
**Sensitivity analysis 2: Matched patients survived the 12-month follow-up period**				
Total all-cause direct medical costs (¥)	63,334	17,920	45,414	<0.001
Inpatient all-cause direct medical costs	38,838	7,679	31,159	<0.001
Outpatient all-cause direct medical costs	24,496	10,241	14,255	<0.001

## Discussion

This was the first study estimating the impact of HK on disease burdens among patients with new-onset CKD in China. HK was a significant predictor of all-cause mortality for patients with CKD, especially during the immediate period after the HK events. When compared with the non-HK cohorts that were matched on the baseline demographic and clinical characteristics, patients with HK incurred more frequent utilization of healthcare services and approximately ¥40,000 greater all-cause total costs driven by hospitalization. Robustness of the results was proved by sensitivity analysis, excluding patients under extreme health status.

The direct clinical and economic consequences of HK have been widely reported in previous studies, regardless of the severity of disease and comorbidity. In a study based on hospitalized patients in Japan, patients with mild, moderate, and severe HK had an increased risk of 3-year mortality, with hazard ratios of 7.6 to 17.7 (16). As for patients with CKD, who are with and without HK, the mortality differences increased dramatically from 51% (55 vs. 4%) in stage 3 to 71% (83 vs. 12%) in stage 5 (16). Patients with CKD in California (RR = 1.5–3.3 for HK cohort) and patients enrolled in Medicare (24.9% in HK cohort vs. 10.4% in non-HK cohort) seem to be less susceptible to higher K^+^ levels (12, 13), due to the wider range of population (not only the hospitalized). The increased mortality associated with HK during the total follow-up period in our study was basically consistent with previous studies, and similar sudden death threats of HK were also proved in patients with CKD in Korea (15), the United States (21) and Italy (22). While, given the patients identified from both outpatient and inpatient claims, the total estimation of mortality was relatively higher in this study, even for patients without HK. This can be explained by the following reasons: first, delays in the diagnosis of CKD may exist in China and some patients, who are newly identified as CKD, are severer than the incident patients. Second, due to the insensitivity of ICD-10 diagnosis for HK (23) and the acknowledged under-reporting of the HK events, the patients included in the HK cohort by definite diagnosis may be those in more serious or acute situations. Finally, for the balanced comparison, the patients extracted into the non-HK cohort may be those who were in poorer health conditions with excessive prevalence of comorbidities. Therefore, the non-HK cohort was also vulnerable to adverse outcomes and under great consumption of healthcare resources.

The CKD severity-adjusted monthly costs in patients with HK were more than twice of those without HK in Medicare ($4,945 vs. 2,137) and commercially insured patients ($7,274 vs. 2,879), respectively (13). Lower HK-associated increased costs were observed in Denmark (€5,077 for a 6-month period) (18) and Japan ($16,969 vs. 4,439 for 1 year) (17). The cost estimations were further lower in our studies for both the cohorts, which was mainly caused by the diversity of healthcare systems, but the difference of costs was consistently 2 to 4 times higher for the HK cohort, and was mainly driven by the higher proportion of hospitalization in patients who had HK, where a steep growth was observed during the first month after the index, which can be probably attributed to specialized medications, monitoring, and other interventions for acute treatment of the index events (14, 16, 17, 24, 25). After the acute episode, the clinical and economic threats of HK were presented, and the difference of costs covered almost all the service and cost categories in our study. This indicated that rather than a causal factor, HK is more likely a biomarker for severity of the underlying disease that suggests the impending deterioration of illness and medical demands. This was supported by previous studies showing that the risk of HK increased linearly as GFR decreased with advancing CKD (2, 26). Therefore, the challenge of HK could be multi-dimensional and the effective treatment options would substantially benefit the patients in need.

Major strength of this study was the application of adjustment methods to estimate the costs. Simple annualization of cost may lead to unfair comparison due to the difference in survival time. The survival-adjusted estimator achieved by the KMSA method provides an effective solution to this challenge (19), and extensive studies showed that the estimators are adequate for practical use and the biases are small even when censoring may occur in the interiors of the intervals (27).This study was also strengthened by the informative description and decomposition of mortality and costs, which provided essential insight about the real-world disease burdens and temporal dynamics of HK, aiming to guide and incent the improvement of disease management.

Limitations of this study were mainly associated with the use of retrospective claims data. Firstly, HK was identified by the ICD-10 code and related Chinese free texts in this study, which may lead to the underestimation of the prevalence. However, to our knowledge, a hyperkalemic event was typically recorded once it occurred in Chinese clinical routine, especially among patients with CKD due to the potential threats. So, identifying HK events based on diagnosis records would be acceptable. Meanwhile, the unavailability of laboratory measures (e.g., eGFR) might also limited the detailed analysis of different subgroups according to disease severity. In addition, while studies suggested that not only the absolute level, but the duration and recurrence of HK and the fluctuation of K^+^ level (even in normal ranges) are associated with poor outcomes (6, 28), this association was not revealed in this study because of the lack of relapsing events. It is possibly explained by the short duration of CKD in the included patients and under-identification of HK. Therefore, future studies based on clear and precise identification of the population are needed in China, which requires a well-designed prospective data collection to capture information on disease severity, anthropometric factors (e.g., body mass index), lifestyle factors (e.g., dietary habit or physical activity), and socioeconomic status. Furthermore, based on the balanced cohorts and the survival-weighted estimator, the effect of HK on costs was indicated by the difference of absolute values without further regression models and adjustment of distribution, which needs to be interpreted with care and more rigorous data analysis should be explored in future studies. Finally, the generalizability of our results may be limited for the whole population in China, as we only included the employees and retirees covered by UEBMI in the municipality of Tianjin. The clinical practice and related costs and medical insurance policies may be heterogeneous across different regions in China and further study based on more representative populations are warranted.

In conclusion, regardless of the existing limitations and required cautions about result interpretation, this study provides essential evidence for the considerable influence of HK on mortality and economic burdens among newly-diagnosed CKD patients in China. This result indicates the necessity of sufficient supervision of K^+^ and the urgent need of effective strategies for the management of serum potassium in China, in order to prevent the acute life-threatening impact of hyperkalemia. Furthermore, there is a need to breakout the dilemma of long-term treatment of underlying diseases, which will ultimately prevent patients from the adverse outcomes and large economic burdens.

## Data Availability Statement

The data analyzed in this study is subject to the following licenses/restrictions: The individual participant data and data dictionary that underlie the results reported in this article will not be shared. Requests to access these datasets should be directed to jingwu@tju.edu.cn.

## Author Contributions

JW and XH contributed to the presented idea and acquisition of data. JZ developed the study design and performed the analysis and interpretation. All authors discussed the results and contributed to the final manuscript.

## Conflict of Interest

The authors declare that the research was conducted in the absence of any commercial or financial relationships that could be construed as a potential conflict of interest.

## Publisher's Note

All claims expressed in this article are solely those of the authors and do not necessarily represent those of their affiliated organizations, or those of the publisher, the editors and the reviewers. Any product that may be evaluated in this article, or claim that may be made by its manufacturer, is not guaranteed or endorsed by the publisher.

## Supplementary Material

Supplementary Tables 1, 2 The baseline characteristics before matching and the monthly costs.
