# Supplementary material for: The Impact of Hyperkalemia on Mortality and Healthcare Resource Utilization Among Patients With Chronic Kidney Disease: A Matched Cohort Study in China
Source: Front Public Health. 2022 Mar 24;10:855395. doi: 10.3389/fpubh.2022.855395 (PMC8987151; doi:10.3389/fpubh.2022.855395)
Supplement: Supplementary Tables 1, 2 — The baseline characteristics before matching and the monthly costs. [file Table_1.DOCX]

|  | | | | | | | | | | | | | | | | | | | | |
| --- | --- | --- | --- | --- | --- | --- | --- | --- | --- | --- | --- | --- | --- | --- | --- | --- | --- | --- | --- | --- |
| **Variables** | | | **HK cohort**  **N=1,015** | | | | | | **non-HK cohort** | | | | | | **p-value** | | | **Standardized difference^1^** | | |
|  |  |  |  |  |  |  |  |  | **N=25,981** | | | | | |  |  |  |  |  |  |
|  |  |  | **Mean/n** | | | **SD/%** | | | **Mean** | | | **SD/%** | | |  |  |  |  |  |  |
| **Demographic characteristics** | | |  | | |  | | |  | | |  | | |  | | |  | | |
| Age at index date [Mean (SD)] | | | 67.3 | | | 14.4 | | | 66.0 | | | 13.3 | | | 0.002 | | | 0.09 | | |
| Female [n(%)] | | | 414 | | | 40.8% | | | 9,380 | | | 36.1% | | | 0.002 | | | 0.10 | | |
| **CCI** | | | 3.9 | | | 2.6 | | | 3.7 | | | 2.2 | | | <0.001 | | | 0.11 | | |
| **CKD duration [month, mean(SD)]** | | | 12.6 | | | 15.3 | | | 16.2 | | | 15.0 | | | <0.001 | | | -0.24 | | |
| **Baseline comorbidity [n(%)]** | | |  | | |  | | |  | | |  | | |  | | |  | | |
| Hypertension | | | 807 | | | 79.5% | | | 20,397 | | | 78.5% | | | 0.446 | | | 0.02 | | |
| Dyslipidemia | | | 468 | | | 46.1% | | | 14,876 | | | 57.3% | | | <0.001 | | | -0.22 | | |
| T2DM | | | 445 | | | 43.8% | | | 14,058 | | | 54.1% | | | <0.001 | | | -0.21 | | |
| Heart failure | | | 210 | | | 20.7% | | | 1,243 | | | 4.8% | | | <0.001 | | | 0.49 | | |
| CVD | | |  | | |  | | |  | | |  | | |  | | |  | | |
| Arrhythmias | | | 285 | | | 28.1% | | | 5,139 | | | 19.8% | | | <0.001 | | | 0.20 | | |
| Angina | | | 212 | | | 20.9% | | | 4,281 | | | 16.5% | | | <0.001 | | | 0.11 | | |
| Myocardial Infarction | | | 99 | | | 9.8% | | | 951 | | | 3.7% | | | <0.001 | | | 0.25 | | |
| Other CVD | | | 399 | | | 39.3% | | | 4,193 | | | 16.1% | | | <0.001 | | | 0.54 | | |
| CBD | | |  | | |  | | |  | | |  | | |  | | |  | | |
| Stroke | | | 315 | | | 31.0% | | | 6,317 | | | 24.3% | | | <0.001 | | | 0.15 | | |
| Transient Ischemic Attack | | | 35 | | | 3.4% | | | 680 | | | 2.6% | | | 0.106 | | | 0.05 | | |
| Other CBD | | | 125 | | | 12.3% | | | 2,733 | | | 10.5% | | | 0.068 | | | 0.06 | | |
| PVD | | | 157 | | | 15.5% | | | 3,957 | | | 15.2% | | | 0.836 | | | 0.01 | | |
| Peptic ulcer | | | 104 | | | 10.2% | | | 2,214 | | | 8.5% | | | 0.054 | | | 0.06 | | |
| COPD | | | 61 | | | 6.0% | | | 633 | | | 2.4% | | | <0.001 | | | 0.18 | | |
| Rheumatic disease | | | 48 | | | 4.7% | | | 674 | | | 2.6% | | | <0.001 | | | 0.11 | | |
| **Baseline treatment [n (%)]** | | |  | | |  | | |  | | |  | | |  | | |  | | |
| Dialysis | | | 29 | | | 2.9% | | | 23 | | | 0.1% | | | <0.001 | | | 0.23 | | |
| RAASi prescription | | |  | | |  | | |  | | |  | | |  | | |  | | |
| ARB | | | 488 | | | 48.1% | | | 12,073 | | | 46.5% | | | 0.313 | | | 0.03 | | |
| MRA | | | 314 | | | 30.9% | | | 1,855 | | | 7.1% | | | <0.001 | | | 0.64 | | |
| ACEi | | | 236 | | | 23.3% | | | 4,377 | | | 16.8% | | | <0.001 | | | 0.16 | | |
| Non-RAASi prescription | | |  | | |  | | |  | | |  | | |  | | |  | | |
| Calcium channel blockers | | | 649 | | | 63.9% | | | 15,547 | | | 59.8% | | | 0.009 | | | 0.08 | | |
| Loop Diuretics | | | 550 | | | 54.2% | | | 5,996 | | | 23.1% | | | <0.001 | | | 0.67 | | |
| NSAID | | | 544 | | | 53.6% | | | 14,753 | | | 56.8% | | | 0.044 | | | -0.06 | | |
| Anti-platelet | | | 480 | | | 47.3% | | | 12,410 | | | 47.8% | | | 0.766 | | | -0.01 | | |
| β-blocker | | | 434 | | | 42.8% | | | 7,759 | | | 29.9% | | | <0.001 | | | 0.27 | | |
| Anti-dyslipidemia | | | 395 | | | 38.9% | | | 11,009 | | | 42.4% | | | 0.029 | | | -0.07 | | |
| Insulin | | | 382 | | | 37.6% | | | 6,567 | | | 25.3% | | | <0.001 | | | 0.27 | | |
| OAD | | | 290 | | | 28.6% | | | 11,315 | | | 43.6% | | | <0.001 | | | -0.32 | | |
| Bronchodilators | | | 281 | | | 27.7% | | | 3,607 | | | 13.9% | | | <0.001 | | | 0.35 | | |
| Anti-hypertension | | | 242 | | | 23.8% | | | 3,257 | | | 12.5% | | | <0.001 | | | 0.30 | | |
| **All-cause healthcare resource utilization and medical costs per patient** | | | | | | | | | | | | | | | | | | | | |
| Number of hospitalizations | | | 1.3 | | | 1.8 | | | 0.4 | | | 1.0 | | | <0.001 | | | 0.62 | | |
| Length of stay | | | 18.7 | | | 31.4 | | | 5.1 | | | 14.7 | | | <0.001 | | | 0.55 | | |
| Number of outpatient visits | | | 36.6 | | | 36.6 | | | 36.6 | | | 31.1 | | | 0.970 | | | 0.00 | | |
| All-cause direct medical cost | | | 33,955 | | | 44,475 | | | 14,428 | | | 22,326 | | | <0.001 | | | 0.55 | | |
| Standard difference was used to compare balance in measured variables between HK and non-HK subjects in the matched sample with that in the unmatched sample. It was defined as d=(X_HK_-X_non-HK_)/(( S^2^_HK_-S^2^_non-HK_)/2)^0.5) for continuous variables (where X denote the sample mean of the covariate in HK and non-HK subjects, and S^2^ denote the sample variance, respectively) and d=(P_HK_-P_non HK_)/((( P_HK_ *(1- P_HK_)+ P_non-HK_ *(1- P_non-HK_))/2)^0.5) for dichotomous variables (where p denote the proportion of the covariate in HK and non-HK subjects). Imbalance defined as absolute value greater than 0.1.  2. Abbreviations: SD, standard deviation; CCI, charlson comorbidity index; CKD, chronic kidney disease; HF, heart failure; T2DM, type 2 diabetes mellitus; CVD, cardiovascular disease; CBD, cerebrovascular disease; PVD, peripheral vascular disease; COPD, chronic obstructive pulmonary disease; ACEi, angiotensin-converting enzyme inhibitors; ARB, angiotensin receptor blockers; MRA, mineralocorticoid receptor antagonists; OAD, oral antidiabetic drugs; NSAID, non-steroidal anti-inflammatory drugs. | | | | | | | | | | | | | | | | | | | | |

**Supplementary Table 1. Baseline characteristics of the HK and non-HK cohorts before propensity score matching**

|  | |  | |  | | | | | | | | | |  |
| --- | --- | --- | --- | --- | --- | --- | --- | --- | --- | --- | --- | --- | --- | --- |
|  | **Total costs** | | | | | | **Inpatient cost** | | | | **Outpatient costs** | | | |
|  | **HK cohort** | | **Non-HK cohort** | | **Difference** | **HK cohort** | | **Non-HK cohort** | **Difference** | **HK cohort** | | **Non-HK cohort** | **Difference** | |
| 1 | 23,630 | | 1,945 | | 21,685 | 22,204 | | 1,032 | 21,173 | 1,426 | | 913 | 513 | |
| 2 | 3,798 | | 1,650 | | 2,148 | 2,510 | | 793 | 1,716 | 1,288 | | 857 | 431 | |
| 3 | 3,357 | | 1,705 | | 1,652 | 2,052 | | 860 | 1,192 | 1,305 | | 844 | 461 | |
| 4 | 3,264 | | 1,469 | | 1,796 | 1,875 | | 649 | 1,227 | 1,389 | | 820 | 569 | |
| 5 | 2,967 | | 1,550 | | 1,416 | 1,543 | | 739 | 805 | 1,423 | | 812 | 611 | |
| 6 | 2,937 | | 1,555 | | 1,382 | 1,534 | | 774 | 760 | 1,402 | | 781 | 622 | |
| 7 | 3,041 | | 1,683 | | 1,358 | 1,666 | | 901 | 764 | 1,375 | | 781 | 594 | |
| 8 | 3,569 | | 1,967 | | 1,602 | 2,064 | | 1,158 | 905 | 1,506 | | 809 | 696 | |
| 9 | 3,218 | | 2,077 | | 1,141 | 1,727 | | 1,297 | 431 | 1,491 | | 781 | 710 | |
| 10 | 3,206 | | 1,756 | | 1,450 | 1,654 | | 943 | 711 | 1,552 | | 813 | 740 | |
| 11 | 3,070 | | 1,798 | | 1,272 | 1,459 | | 1,031 | 427 | 1,612 | | 767 | 845 | |
| 12 | 3,153 | | 1,576 | | 1,577 | 1,567 | | 803 | 765 | 1,586 | | 773 | 812 | |

**Supplementary Table 2. The monthly all-cause direct medical costs in the 12-month follow-up period (**¥)
